# Idiopathic orbital inflammatory disease - a diagnostic dilemma

**DOI:** 10.22336/rjo.2024.83

**Published:** 2024

**Authors:** Sarita Lobo, Geover Joslen Lobo

**Affiliations:** 1 Department of Ophthalmology, Father Muller Medical College, Mangalore, India; 2 Department of Neurosurgery, Father Muller Medical College, Mangalore, India

**Keywords:** idiopathic orbital inflammation, proptosis, pseudotumor, OID = Orbital Inflammatory Disease, h/o = history of, PL = perception of light, NRTL = normally reactive to light, NSOI = nonspecific orbital inflammation, IOI = idiopathic orbital inflammation

## Abstract

Orbital pseudotumor is nonspecific orbital inflammation (NSOI). It is a benign, non-infectious, space-occupying inflammatory lesion of the orbit. NSOI can affect various tissues in the orbit, such as the lacrimal gland and extraocular muscles. The most common classification is based on clinical presentation. Bacteria, viruses, fungi, or parasites can cause infectious orbital inflammation. It is a diagnosis of exclusion after ruling out inflammatory, infectious, and neoplastic causes. We present a case of a male in his sixties who presented with progressive pain, swelling, blurry vision, and forward protrusion of his right eye. He had no history of trauma or recent illness. His general physical and systemic examination was within normal limits. His ocular examination showed eyelid edema, erythema, eccentric proptosis, and a mature cataract in his right eye. His left eye showed a lenticular opacity. CT orbit revealed a homogenous isodense lesion observed without any globe distortion. A diagnosis of orbital pseudotumor was made. The patient was treated with oral corticosteroids, and an excision biopsy was done, resulting in symptomatic improvement and regression of inflammation at follow-up. In complex cases of inflammatory orbital pseudotumor, particularly those with granulomatous inflammation, some initial success has occurred with monoclonal antibodies against tumor necrosis factor (TNF)-alpha or with lymphocyte depletion using rituximab. Our patient, however, responded well to an excision biopsy and a course of oral steroids.

## Introduction

Orbital Inflammatory Disease (OID) is a heterogeneous group of conditions characterized by inflammation of the orbital tissues. It encompasses a broad spectrum of diseases, ranging from self-limiting conditions to chronic, progressive disorders with significant visual morbidity. We discussed a case report on orbital inflammatory disease and provided an overview of the literature review.

## Case report

**Fig. 1:**
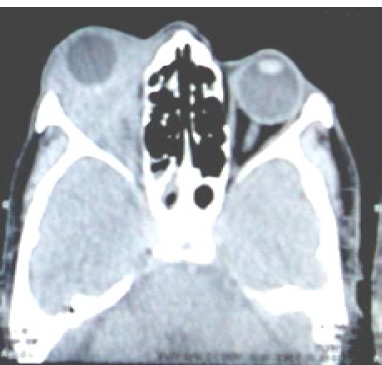
CT ORBIT - Plain and with contrast - Homogenous isodense lesion observed without any distortion of the globe

**Fig. 2:**
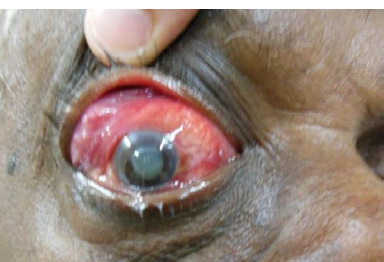
Right eye with superotemporal mass lesion with eccentric proptosis

**Fig. 3:**
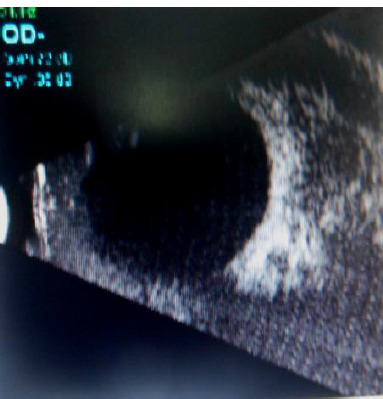
USG ORBIT - Retina attached ill-defined heterogeneous lesion observed behind the globe in the superotemporal aspect and around the optic nerve

A male patient in his sixties presented with chief complaints of protrusion of the right eyeball for 5 years, gradually increasing in size, with associated pain and decreasing vision for the past 1 month. No h/o trauma. No h/o fever/loss of weight and appetite. Examination of the right eye - Vision - PL+, with periocular hyperpigmentation, eccentric proptosis (pushed down and slightly in) with an inability to insinuate superior, lateral aspect of the orbit. Resistance to retropulsion was present. No bruit/pulsations. Abduction and elevation were restricted. Anterior segment examination showed conjunctival chemosis and congestion. A swelling was observed in the superotemporal aspect of the lacrimal gland region. The cornea was clear. The anterior chamber was shallow. The pupil was 6 mm NRTL, lens - mature cataract, fundus - no view (**Fig. 1, 2**). Left eye examination showed visual acuity -6/18. Lenticular opacity was present. The rest of the IOP examination was within normal limits. Blood investigations were within normal limits. Ocular B scan showed retina attached. An ill-defined heterogeneous lesion was observed behind the globe in the superotemporal aspect and around the optic nerve **Fig. 3**. Abdominal USG was within normal limits. Differential diagnosis of idiopathic orbital inflammatory disease and lymphoproliferative disease was considered.

## Results

**Fig. 4:**
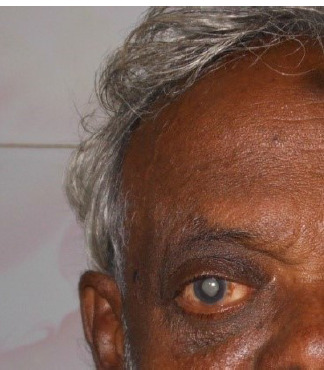
Dramatic improvement with steroids

**Fig. 5:**
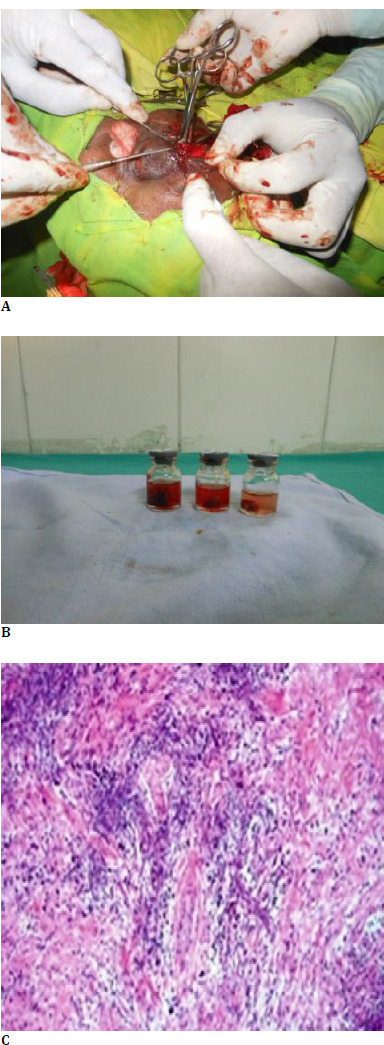
**A-C** HPE of the lesion - Fibrosis and granulomatous reaction with inflammatory infiltrates

A course of oral steroids 1 mg/kg was given, after which a biopsy of the lesion was done. Histopathology confirmed the diagnosis of orbital inflammatory disease (**Fig. 4**). Following a course of steroids and surgical excision biopsy, the lesion was utterly resolved (**Fig. 5 A-C**).

## Discussion

Orbital inflammatory disease can be classified based on various criteria, including etiology, clinical presentation, and histopathology. The most common classification is based on clinical presentation. Idiopathic orbital inflammation (IOI), the most common type, is characterized by nonspecific orbit inflammation. Bacteria, viruses, fungi, or parasites cause infectious orbital inflammation. Allergic orbital inflammation is associated with allergic conditions like eosinophilic granuloma. Granulomatous orbital inflammation includes conditions like sarcoidosis, Wegener’s granulomatosis, and IgG4-related disease. Orbital pseudotumor is a benign inflammatory mass of unknown etiology. Clinical manifestations of OID vary widely depending on the underlying cause and extent of inflammation. Common symptoms include proptosis (bulging of the eye), pain, redness, swelling, diplopia (double vision), and decreased visual acuity. Apical or posterior involvement is less common and is associated with a poor visual outcome [1]. Clinically, apical IOI presents with orbital pain, restricted eye movement, visual loss, and minimal proptosis [2]. Inflammatory lesions of the orbital apex may extend intracranially through the superior orbital fissure, optic canal, and inferior orbital fissure. Similar symptoms and physical findings occur with inflammatory orbital pseudotumor and orbital infection. Still, there is no history of trauma or adjacent focus of infection (e.g., sinusitis) with inflammatory orbital pseudotumor. Neuroimaging with CT or MRI is required. A useful imaging feature in distinguishing an infection from noninfectious inflammation is the presence of adjacent sinus involvement in orbital infection. A biopsy may be used for chronic or recurrent disease to find evidence of an underlying medical condition. In thyroid eye disease, tendon-sparing enlargement of the inferior and medial rectus muscles is common [3,4]. Treatment of inflammatory orbital pseudotumor depends on the type of inflammatory response and may include oral corticosteroids, radiation therapy, and one of several immunomodulating medications. In complex cases of inflammatory orbital pseudotumor, particularly those with granulomatous inflammation, some initial success has occurred with monoclonal antibodies against tumor necrosis factor (TNF)-alpha or with lymphocyte depletion using rituximab [5,6]. Our patient, however, responded well to an excision biopsy and a course of oral steroids.

## Conclusion

Idiopathic orbital inflammatory disease is a non-neoplastic, non-infectious space-occupying lesion, usually in the third to fifth decades. It presents as periorbital redness, swelling, congestive proptosis, and ophthalmoplegia. If severe, systemic steroids, 60-80 mg/day, which is the mainstay of therapy, are administered, radiotherapy/antimetabolites are given in resistant cases. Biopsy is done in persistent instances to rule out neoplasia.
